# Crystal structure of ethyl *N*-(1,5-dimethyl-3-oxo-2-phenyl-2,3-di­hydro-1*H*-pyrazol-4-yl)carbamate

**DOI:** 10.1107/S2056989015006106

**Published:** 2015-03-28

**Authors:** Muhammad Danish, Muhammad Nawaz Tahir, Uzma Anwar, Muhammad Asam Raza

**Affiliations:** a Department of Chemistry, Institute of Natural Sciences, University of Gujrat, Gujrat 50700, Pakistan; b Department of Physics, University of Sargodha, Sargodha, Punjab, Pakistan

**Keywords:** crystal structure, di­hydro­pyrazole, ethyl ester, carbamate, hydrogen bonding, π–π stacking inter­actions

## Abstract

In the title compound, C_14_H_17_N_3_O_3_, the dihedral angle between the benzene ring and the five-membered di­hydro­pyrazole ring is 52.26 (9)°. The ethyl ester group is approximately planar (r.m.s. deviation 0.0568 Å) and subtends an angle 67.73 (8)° to the pyrazole ring. In the crystal, molecules are linked by pairs of N—H⋯O hydrogen bonds, forming inversion dimers with an *R*_2_^2^(10) ring motif. Weaker C—H⋯O contacts link these dimers into a three-dimensional network of mol­ecules stacked along the *a*-axis direction. Offset π–π stacking inter­actions between the benzene rings [centroid-to-centroid distance = 3.8832 (12) Å] further stabilize the crystal packing.

## Related literature   

For related structures see: Li *et al.* (2013 ▸).
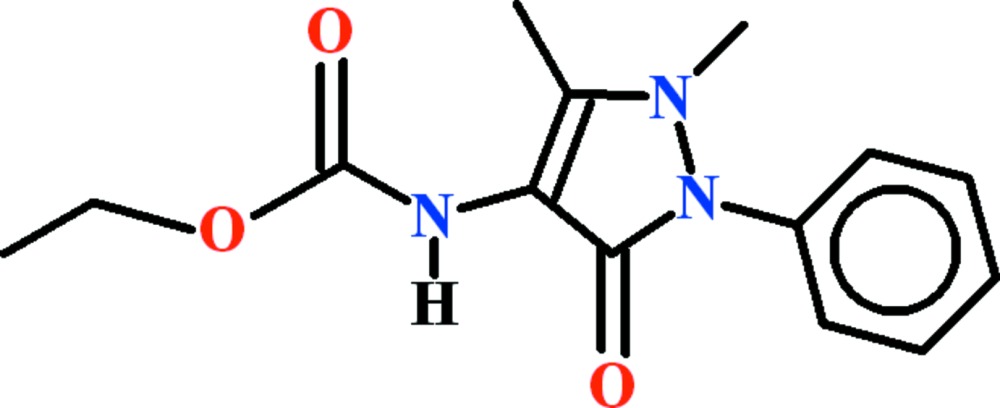


## Experimental   

### Crystal data  

C_14_H_17_N_3_O_3_
*M* *_r_* = 275.30
Monoclinic, 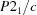

*a* = 8.2100 (4) Å
*b* = 11.4137 (6) Å
*c* = 15.1594 (8) Å
β = 93.403 (3)°
*V* = 1418.03 (13) Å^3^
*Z* = 4
Mo *K*α radiation
μ = 0.09 mm^−1^
*T* = 296 K
0.38 × 0.34 × 0.20 mm

### Data collection  

Bruker Kappa APEXII CCD diffractometer
Absorption correction: multi-scan (*SADABS*; Bruker, 2005 ▸) *T*_min_ = 0.968, *T*_max_ = 0.983
11712 measured reflections
3098 independent reflections
2368 reflections with *I* > 2σ(*I*)
*R*_int_ = 0.022

### Refinement  

*R*[*F* ^2^ > 2σ(*F* ^2^)] = 0.043
*wR*(*F* ^2^) = 0.127
*S* = 1.06
3098 reflections
185 parameters
H-atom parameters constrained
Δρ_max_ = 0.19 e Å^−3^
Δρ_min_ = −0.17 e Å^−3^

### 

Data collection: *APEX2* (Bruker, 2007 ▸); cell refinement: *SAINT* (Bruker, 2007 ▸); data reduction: *SAINT*; program(s) used to solve structure: *SHELXS97* (Sheldrick, 2008 ▸); program(s) used to refine structure: *SHELXL2014*/6 (Sheldrick, 2015 ▸); molecular graphics: *ORTEP-3 for Windows* (Farrugia, 2012 ▸) and *PLATON* (Spek, 2009 ▸); software used to prepare material for publication: *WinGX* (Farrugia, 2012 ▸) and *PLATON*.

## Supplementary Material

Crystal structure: contains datablock(s) global, I. DOI: 10.1107/S2056989015006106/sj5447sup1.cif

Structure factors: contains datablock(s) I. DOI: 10.1107/S2056989015006106/sj5447Isup2.hkl

Supporting information file. DOI: 10.1107/S2056989015006106/sj5447Isup3.cml

**Fig. 1.**
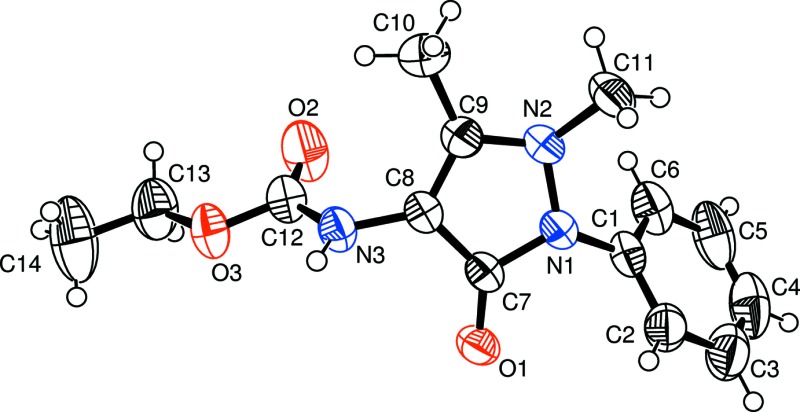
View of the asymmetric unit of title compound with the atom-numbering scheme. Displacement ellipsoids are drawn at the 50% probability level. H atoms are shown as small circles of arbitrary radii.

**Fig. 2.**
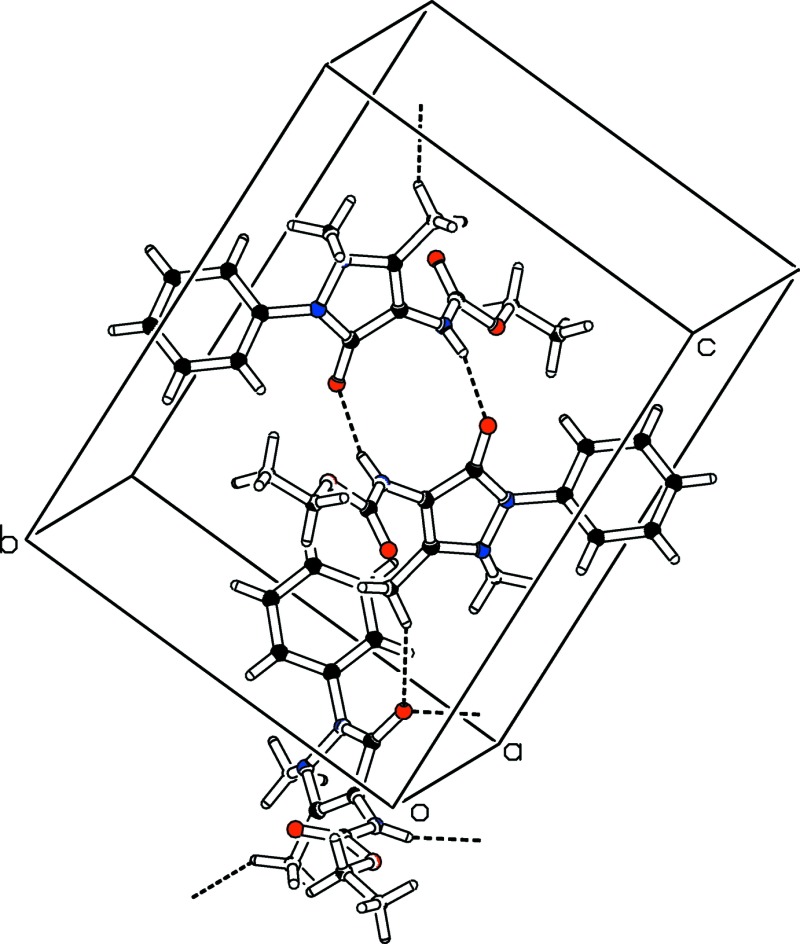
The crystal packing of (I), viewed along the a axis, with hydrogen bonds shown as dashed lines.

. DOI: 10.1107/S2056989015006106/sj5447fig1.tif
View of the asymmetric unit of title compound with the atom-numbering scheme. Displacement ellipsoids are drawn at the 50% probability level. H atoms are shown as small circles of arbitrary radii.

a . DOI: 10.1107/S2056989015006106/sj5447fig2.tif
The crystal packing of (I), viewed along the *a* axis, with hydrogen bonds shown as dashed lines.

CCDC reference: 1056157

Additional supporting information:  crystallographic information; 3D view; checkCIF report

## Figures and Tables

**Table 1:** Hydrogen-bond geometry (, )

*D*H*A*	*D*H	H*A*	*D* *A*	*D*H*A*
N3H3*A*O1^i^	0.86	1.99	2.8223(17)	164
C10H10*A*O1^ii^	0.96	2.56	3.343(2)	139
C11H11*B*O2^iii^	0.96	2.66	3.576(2)	161

Symmetry codes: (i) 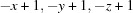; (ii) 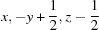; (iii) 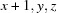.

## supplementary crystallographic information

### S1. Comment

The title compound (I), (Fig. 1) has been synthesized to examine its DNA binding potential and anti-microbial activity. The crystal structures of two closely related compounds, methyl (1,5-dimethyl-3-oxo-2-phenyl-2,3-dihydro-1*H*- pyrazol-4-yl)carbamate and methyl (1,5-dimethyl-3-oxo-2-phenyl-2,3-dihydro-1*H*-pyrazol-4-yl)methylcarbamate monohydrate have been reported previously (Li *et al.*, 2013).

In (I), the C1—C6 benzene ring and dihydropyrazol-3-one system, O1/N1/N2/N3/C7—C9 are planar with *r.m.s.* deviations of 0.0037 and 0.0398 Å, respectively. The dihedral angle between the two systems is 53.64 (5)°. The ethyl ester group, C12/C13/C14/O2/O3, is also planar (*r.m.s.* deviation 0.0568 Å) and subtends an angle 67.73 (8)° to the pyrazole ring. In the crystal structure classical N3—H3A···O1 hydrogen bonds form inversion dimers (Table 1, Fig. 2) with *R*_2_^2^(10) ring motifs. Weaker C10–H10A···O1 and C11–H11B···O2 contacts link these dimers into a three dimensional network of molecules stacked along the a axis direction. Offset π···π stacking interactions between the benzene rings with Cg2···Cg2^i^ = 3.8832 (12) Å further stabilise the crystal packing (Cg2 is the centroid of the C1—C6 benzene ring; i = 2 - *x*, -*y*, 1 - *z*).

### S2. Experimental

4-aminoantipyrene (2 g, 9.84 mmole) was dissolved in 50 ml dimethyl formamide and anhydrous potassium carbonate (0.68 g, 4.92 mmole) was added with continuous stirring over 50 minutes. Ethyl chloroformate (1.3 ml) was added drop-wise with stirring over three minutes at 323 K. TLC monitoring showed the reaction to be complete in almost 4 h. The reaction mixture was then cooled to room temperature and transferred to crushed ice leading to the formation of a precipitate. This was separated by filtration and recrystallized from aqueous ethanol to yield (I) as light yellow plates.

Melting point: 477–479 K.

### S3. Refinement

The H-atoms were positioned geometrically (N–H = 0.86, C–H = 0.93–0.97 Å) and refined as riding with *U*_iso_(H) = 1.2U_eq_(C, N).

### Crystal data

**Table:**

C_14_H_17_N_3_O_3_	*F*(000) = 584
*M**_r_* = 275.30	*D*_x_ = 1.290 Mg m^−^^3^
Monoclinic, *P*2_1_/*c*	Mo *K*α radiation, λ = 0.71073 Å
*a* = 8.2100 (4) Å	Cell parameters from 2368 reflections
*b* = 11.4137 (6) Å	θ = 2.2–27.0°
*c* = 15.1594 (8) Å	µ = 0.09 mm^−^^1^
β = 93.403 (3)°	*T* = 296 K
*V* = 1418.03 (13) Å^3^	Plate, light yellow
*Z* = 4	0.38 × 0.34 × 0.20 mm

### Data collection

**Table:**

Bruker Kappa APEXII CCD diffractometer	3098 independent reflections
Radiation source: fine-focus sealed tube	2368 reflections with *I* > 2σ(*I*)
Graphite monochromator	*R*_int_ = 0.022
Detector resolution: 7.80 pixels mm^-1^	θ_max_ = 27.0°, θ_min_ = 2.2°
ω scans	*h* = −10→10
Absorption correction: multi-scan (*SADABS*; Bruker, 2005)	*k* = −14→13
*T*_min_ = 0.968, *T*_max_ = 0.983	*l* = −19→19
11712 measured reflections	

### Refinement

**Table:**

Refinement on *F*^2^	Secondary atom site location: difference Fourier map
Least-squares matrix: full	Hydrogen site location: inferred from neighbouring sites
*R*[*F*^2^ > 2σ(*F*^2^)] = 0.043	H-atom parameters constrained
*wR*(*F*^2^) = 0.127	*w* = 1/[σ^2^(*F*_o_^2^) + (0.0592*P*)^2^ + 0.2848*P*] where *P* = (*F*_o_^2^ + 2*F*_c_^2^)/3
*S* = 1.06	(Δ/σ)_max_ < 0.001
3098 reflections	Δρ_max_ = 0.19 e Å^−^^3^
185 parameters	Δρ_min_ = −0.17 e Å^−^^3^
0 restraints	Extinction correction: *SHELXL*, Fc^*^=kFc[1+0.001xFc^2^λ^3^/sin(2θ)]^-1/4^
Primary atom site location: structure-invariant direct methods	Extinction coefficient: 0.027 (3)

### Special details

**Table:**

Geometry. All e.s.d.'s (except the e.s.d. in the dihedral angle between two l.s. planes) are estimated using the full covariance matrix. The cell e.s.d.'s are taken into account individually in the estimation of e.s.d.'s in distances, angles and torsion angles; correlations between e.s.d.'s in cell parameters are only used when they are defined by crystal symmetry. An approximate (isotropic) treatment of cell e.s.d.'s is used for estimating e.s.d.'s involving l.s. planes.
Refinement. Refinement of *F*^2^ against ALL reflections. The weighted *R*-factor *wR* and goodness of fit *S* are based on *F*^2^, conventional *R*-factors *R* are based on *F*, with *F* set to zero for negative *F*^2^. The threshold expression of *F*^2^ > σ(*F*^2^) is used only for calculating *R*-factors(gt) *etc*. and is not relevant to the choice of reflections for refinement. *R*-factors based on *F*^2^ are statistically about twice as large as those based on *F*, and *R*-factors based on ALL data will be even larger.

### Fractional atomic coordinates and isotropic or equivalent isotropic displacement parameters (Å^2^)

**Table:**

	*x*	*y*	*z*	*U*_iso_*/*U*_eq_	
O1	0.54493 (13)	0.33953 (9)	0.54004 (6)	0.0503 (3)	
O2	0.13321 (16)	0.32771 (12)	0.33957 (11)	0.0819 (5)	
O3	0.09391 (13)	0.51504 (10)	0.38192 (8)	0.0617 (3)	
N1	0.65770 (15)	0.23072 (11)	0.42996 (8)	0.0458 (3)	
N2	0.62265 (16)	0.21412 (12)	0.33892 (8)	0.0510 (3)	
N3	0.34531 (15)	0.44615 (11)	0.38501 (8)	0.0480 (3)	
H3A	0.3728	0.5172	0.3977	0.058*	
C1	0.72550 (18)	0.13738 (13)	0.48290 (10)	0.0472 (4)	
C2	0.8305 (2)	0.16528 (17)	0.55395 (11)	0.0592 (4)	
H2	0.8595	0.2428	0.5653	0.071*	
C3	0.8922 (3)	0.0764 (2)	0.60812 (14)	0.0791 (6)	
H3	0.9628	0.0945	0.6565	0.095*	
C4	0.8508 (3)	−0.0376 (2)	0.59139 (18)	0.0851 (7)	
H4	0.8924	−0.0967	0.6285	0.102*	
C5	0.7481 (3)	−0.06516 (17)	0.52008 (19)	0.0841 (7)	
H5	0.7216	−0.1431	0.5085	0.101*	
C6	0.6830 (2)	0.02230 (15)	0.46480 (14)	0.0639 (5)	
H6	0.6122	0.0038	0.4166	0.077*	
C7	0.55368 (17)	0.31534 (12)	0.46080 (9)	0.0406 (3)	
C8	0.46691 (17)	0.35940 (13)	0.38352 (9)	0.0430 (3)	
C9	0.51590 (19)	0.30017 (14)	0.31216 (10)	0.0487 (4)	
C10	0.4694 (3)	0.3221 (2)	0.21696 (12)	0.0755 (6)	
H10A	0.4626	0.2489	0.1858	0.113*	
H10B	0.5502	0.3709	0.1921	0.113*	
H10C	0.3654	0.3607	0.2117	0.113*	
C11	0.7583 (2)	0.17664 (16)	0.28711 (12)	0.0621 (5)	
H11A	0.7971	0.1016	0.3078	0.093*	
H11B	0.8452	0.2329	0.2937	0.093*	
H11C	0.7217	0.1709	0.2259	0.093*	
C12	0.1866 (2)	0.42031 (14)	0.36699 (11)	0.0528 (4)	
C13	−0.0815 (2)	0.4956 (2)	0.37242 (17)	0.0818 (6)	
H13A	−0.1108	0.4609	0.3152	0.098*	
H13B	−0.1145	0.4424	0.4179	0.098*	
C14	−0.1628 (3)	0.6069 (3)	0.3803 (2)	0.1237 (11)	
H14A	−0.2786	0.5946	0.3798	0.186*	
H14B	−0.1381	0.6563	0.3316	0.186*	
H14C	−0.1259	0.6438	0.4348	0.186*	

### Atomic displacement parameters (Å^2^)

**Table:**

	*U*^11^	*U*^22^	*U*^33^	*U*^12^	*U*^13^	*U*^23^
O1	0.0619 (7)	0.0475 (6)	0.0425 (6)	0.0067 (5)	0.0099 (5)	−0.0044 (4)
O2	0.0552 (7)	0.0589 (9)	0.1307 (13)	−0.0026 (6)	−0.0010 (8)	−0.0187 (8)
O3	0.0453 (6)	0.0558 (7)	0.0849 (8)	0.0115 (5)	0.0113 (6)	0.0010 (6)
N1	0.0493 (7)	0.0440 (7)	0.0445 (7)	0.0090 (6)	0.0050 (5)	−0.0052 (5)
N2	0.0517 (7)	0.0558 (8)	0.0458 (7)	0.0106 (6)	0.0067 (6)	−0.0112 (6)
N3	0.0459 (7)	0.0395 (7)	0.0586 (8)	0.0054 (5)	0.0037 (6)	−0.0030 (5)
C1	0.0421 (7)	0.0439 (8)	0.0568 (9)	0.0089 (6)	0.0134 (6)	0.0019 (7)
C2	0.0558 (9)	0.0613 (11)	0.0607 (10)	0.0102 (8)	0.0043 (8)	0.0035 (8)
C3	0.0691 (12)	0.0960 (17)	0.0724 (12)	0.0247 (12)	0.0054 (10)	0.0187 (11)
C4	0.0744 (14)	0.0786 (15)	0.1049 (17)	0.0307 (12)	0.0268 (13)	0.0369 (13)
C5	0.0725 (13)	0.0453 (11)	0.138 (2)	0.0119 (10)	0.0371 (14)	0.0176 (12)
C6	0.0542 (10)	0.0488 (10)	0.0901 (13)	0.0032 (8)	0.0156 (9)	−0.0036 (9)
C7	0.0411 (7)	0.0350 (7)	0.0464 (8)	−0.0002 (6)	0.0086 (6)	−0.0038 (6)
C8	0.0423 (7)	0.0396 (8)	0.0476 (8)	0.0030 (6)	0.0067 (6)	−0.0019 (6)
C9	0.0472 (8)	0.0521 (9)	0.0470 (8)	0.0031 (7)	0.0029 (6)	−0.0056 (6)
C10	0.0805 (13)	0.0979 (16)	0.0474 (10)	0.0178 (11)	−0.0019 (9)	−0.0074 (9)
C11	0.0611 (10)	0.0664 (11)	0.0605 (10)	0.0110 (9)	0.0174 (8)	−0.0146 (8)
C12	0.0487 (8)	0.0479 (9)	0.0622 (10)	0.0056 (7)	0.0067 (7)	0.0021 (7)
C13	0.0470 (10)	0.0869 (15)	0.1121 (17)	0.0083 (10)	0.0091 (10)	0.0007 (13)
C14	0.0581 (13)	0.105 (2)	0.211 (3)	0.0252 (14)	0.0337 (17)	0.016 (2)

### Geometric parameters (Å, º)

**Table:**

O1—C7	1.2387 (17)	C4—H4	0.9300
O2—C12	1.208 (2)	C5—C6	1.390 (3)
O3—C12	1.3489 (19)	C5—H5	0.9300
O3—C13	1.456 (2)	C6—H6	0.9300
N1—C7	1.3886 (18)	C7—C8	1.426 (2)
N1—N2	1.4057 (17)	C8—C9	1.357 (2)
N1—C1	1.4269 (19)	C9—C10	1.492 (2)
N2—C9	1.362 (2)	C10—H10A	0.9600
N2—C11	1.4645 (19)	C10—H10B	0.9600
N3—C12	1.348 (2)	C10—H10C	0.9600
N3—C8	1.4072 (18)	C11—H11A	0.9600
N3—H3A	0.8600	C11—H11B	0.9600
C1—C2	1.376 (2)	C11—H11C	0.9600
C1—C6	1.383 (2)	C13—C14	1.443 (3)
C2—C3	1.382 (3)	C13—H13A	0.9700
C2—H2	0.9300	C13—H13B	0.9700
C3—C4	1.365 (3)	C14—H14A	0.9600
C3—H3	0.9300	C14—H14B	0.9600
C4—C5	1.367 (4)	C14—H14C	0.9600
			
C12—O3—C13	115.20 (15)	C9—C8—C7	108.79 (13)
C7—N1—N2	109.28 (11)	N3—C8—C7	123.72 (12)
C7—N1—C1	123.86 (12)	C8—C9—N2	109.74 (13)
N2—N1—C1	120.12 (12)	C8—C9—C10	128.08 (15)
C9—N2—N1	106.61 (11)	N2—C9—C10	122.17 (14)
C9—N2—C11	123.19 (14)	C9—C10—H10A	109.5
N1—N2—C11	116.61 (13)	C9—C10—H10B	109.5
C12—N3—C8	121.39 (13)	H10A—C10—H10B	109.5
C12—N3—H3A	119.3	C9—C10—H10C	109.5
C8—N3—H3A	119.3	H10A—C10—H10C	109.5
C2—C1—C6	120.94 (16)	H10B—C10—H10C	109.5
C2—C1—N1	118.21 (15)	N2—C11—H11A	109.5
C6—C1—N1	120.81 (15)	N2—C11—H11B	109.5
C1—C2—C3	119.05 (19)	H11A—C11—H11B	109.5
C1—C2—H2	120.5	N2—C11—H11C	109.5
C3—C2—H2	120.5	H11A—C11—H11C	109.5
C4—C3—C2	120.7 (2)	H11B—C11—H11C	109.5
C4—C3—H3	119.6	O2—C12—N3	125.98 (15)
C2—C3—H3	119.6	O2—C12—O3	124.21 (15)
C3—C4—C5	120.1 (2)	N3—C12—O3	109.79 (14)
C3—C4—H4	120.0	C14—C13—O3	108.53 (19)
C5—C4—H4	120.0	C14—C13—H13A	110.0
C4—C5—C6	120.6 (2)	O3—C13—H13A	110.0
C4—C5—H5	119.7	C14—C13—H13B	110.0
C6—C5—H5	119.7	O3—C13—H13B	110.0
C1—C6—C5	118.6 (2)	H13A—C13—H13B	108.4
C1—C6—H6	120.7	C13—C14—H14A	109.5
C5—C6—H6	120.7	C13—C14—H14B	109.5
O1—C7—N1	123.62 (13)	H14A—C14—H14B	109.5
O1—C7—C8	131.54 (13)	C13—C14—H14C	109.5
N1—C7—C8	104.82 (12)	H14A—C14—H14C	109.5
C9—C8—N3	127.41 (14)	H14B—C14—H14C	109.5
			
C7—N1—N2—C9	9.01 (16)	C12—N3—C8—C9	67.6 (2)
C1—N1—N2—C9	161.31 (13)	C12—N3—C8—C7	−108.89 (18)
C7—N1—N2—C11	151.05 (14)	O1—C7—C8—C9	−177.01 (16)
C1—N1—N2—C11	−56.66 (19)	N1—C7—C8—C9	1.68 (16)
C7—N1—C1—C2	−64.2 (2)	O1—C7—C8—N3	0.0 (3)
N2—N1—C1—C2	147.71 (14)	N1—C7—C8—N3	178.73 (13)
C7—N1—C1—C6	113.91 (17)	N3—C8—C9—N2	−172.97 (14)
N2—N1—C1—C6	−34.2 (2)	C7—C8—C9—N2	3.95 (18)
C6—C1—C2—C3	−0.7 (2)	N3—C8—C9—C10	8.5 (3)
N1—C1—C2—C3	177.40 (15)	C7—C8—C9—C10	−174.57 (18)
C1—C2—C3—C4	0.4 (3)	N1—N2—C9—C8	−7.90 (17)
C2—C3—C4—C5	0.5 (3)	C11—N2—C9—C8	−146.82 (15)
C3—C4—C5—C6	−1.0 (3)	N1—N2—C9—C10	170.73 (16)
C2—C1—C6—C5	0.2 (2)	C11—N2—C9—C10	31.8 (3)
N1—C1—C6—C5	−177.87 (15)	C8—N3—C12—O2	−8.1 (3)
C4—C5—C6—C1	0.7 (3)	C8—N3—C12—O3	173.51 (13)
N2—N1—C7—O1	172.30 (14)	C13—O3—C12—O2	6.8 (3)
C1—N1—C7—O1	21.3 (2)	C13—O3—C12—N3	−174.78 (15)
N2—N1—C7—C8	−6.52 (16)	C12—O3—C13—C14	−173.5 (2)
C1—N1—C7—C8	−157.55 (14)		

### Hydrogen-bond geometry (Å, º)

**Table:**

*D*—H···*A*	*D*—H	H···*A*	*D*···*A*	*D*—H···*A*
N3—H3*A*···O1^i^	0.86	1.99	2.8223 (17)	164
C10—H10*A*···O1^ii^	0.96	2.56	3.343 (2)	139
C11—H11*B*···O2^iii^	0.96	2.66	3.576 (2)	161

Symmetry codes: (i) −*x*+1, −*y*+1, −*z*+1; (ii) *x*, −*y*+1/2, *z*−1/2; (iii) *x*+1, *y*, *z*.
