# Supplementary figures and images for: Crystal structure of ethyl N-(1,5-dimethyl-3-oxo-2-phenyl-2,3-di­hydro-1H-pyrazol-4-yl)carbamate
Source: Acta Crystallogr E Crystallogr Commun. 2015 Mar 28;71(Pt 4):o267. doi: 10.1107/S2056989015006106 (PMC4438802; doi:10.1107/S2056989015006106)

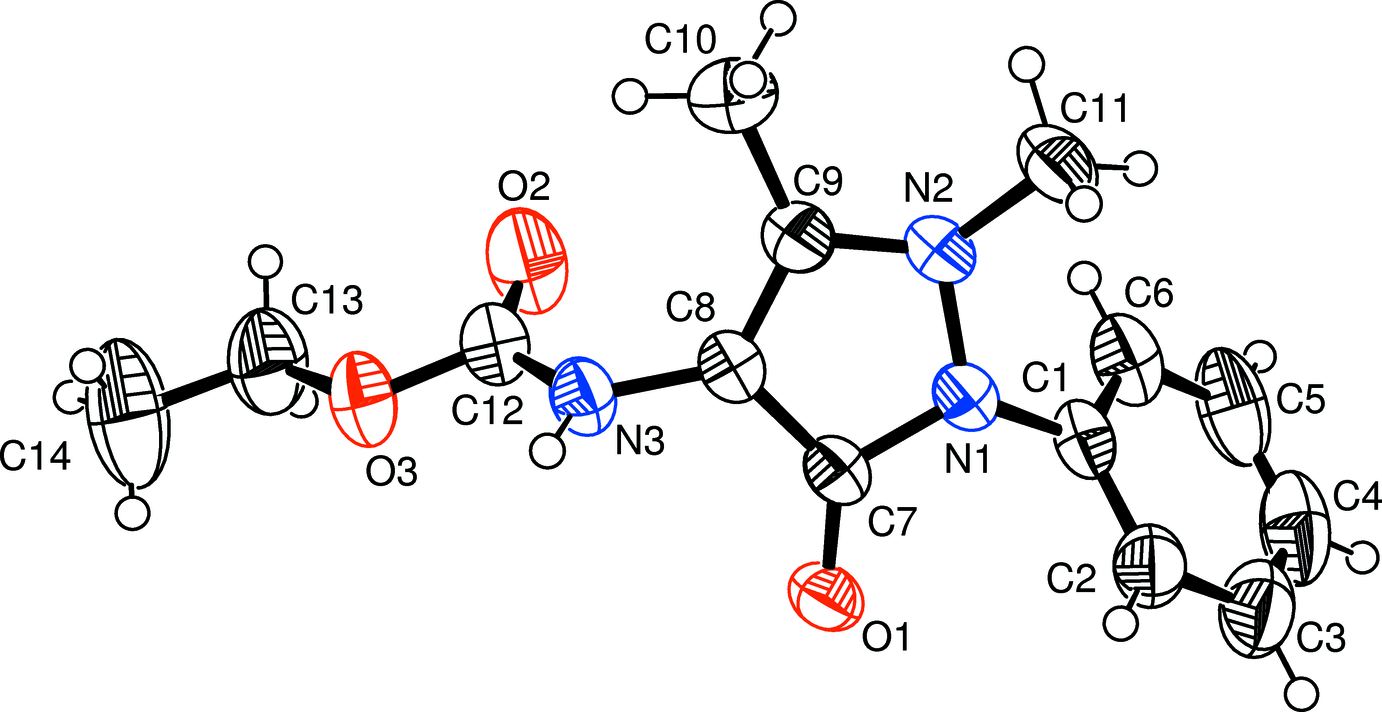

Supplement: Supplementary file 4 [file e-71-0o267-fig1.tif]

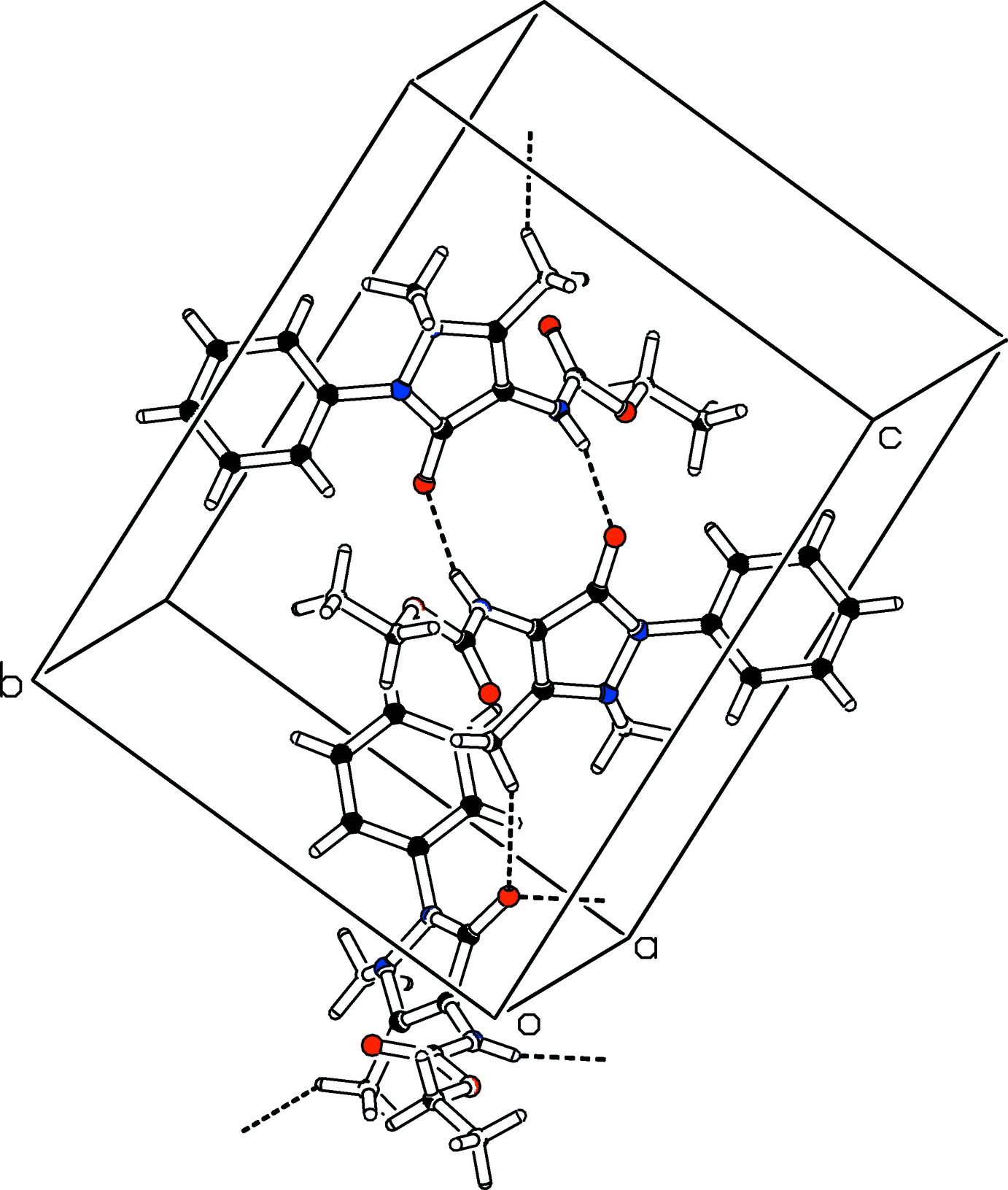

Supplement: Supplementary file 5 [file e-71-0o267-fig2.tif]
